# A blinded-endpoint, randomized controlled trial of Sanyrene with natural active ingredient for prophylaxis of radiation dermatitis in patients receiving radiotherapy

**DOI:** 10.1186/s13014-023-02363-9

**Published:** 2023-10-27

**Authors:** Xiaoli Long, Jing Guo, Yutian Yin, Ming Cheng, Xiao Zhang, Jing Zhang, Pengyuan Wang, Jian Zang, Lina Zhao

**Affiliations:** Department of Radiation Oncology, Xijing Hospital, Fourth Military Medical University, Xi’an, 710032 Shaanxi China

**Keywords:** Radiation dermatitis, Radiotherapy, Sanyrene, Randomized controlled trial

## Abstract

**Background:**

Randomized controlled study was conducted to evaluate the efficacy of Sanyrene® vs. control intervention (DaBao®, a complex of hyaluronic acid and Vitamin E) for acute radiation dermatitis in patients receiving radiotherapy.

**Methods:**

Patients with breast cancer or head and neck cancer undergoing radiotherapy (≥ 50 Gy) were eligible. Participants were randomly assigned to either Sanyrene arm or control intervention arm in a ratio of 1:1. The primary endpoint was incidence rate of ≥ grade 2 radiation induced dermatitis. (Trial Registration: ChiCTR2100050910, registration date: 9/7/2021)

**Results:**

A total of 102 eligible patients were randomly assigned into the study. The rate of ≥ grade 2 radiation dermatitis was 22% in Sanyrene group, as compared with 67.3% in the control intervention group (*P*<0.001). The incidence of grade 3 radiation dermatitis was 20.4% and 8.0% in control intervention group and Sanyrene group, respectively (*P* = 0.076). Patients in Sanyrene group had a longer median time to reach ≥ grade 2 radiation dermatitis compared to these in control intervention group, with hazard ratio of 0.231 (95%CI:0.116–0.458, *p* < 0.001). Mean score of SD-16 were much higher in control intervention group than Sanyrene group at end of radiotherapy (25 vs.8.3), 2 weeks after radiotherapy (22.9 vs. 0.5) and 4 weeks after radiotherapy (4.2 vs.0), with significantly statistical difference between two groups.

**Conclusions:**

This trial suggests that Sanyrene is effective on preventing serious radiation dermatitis and improving skin related quality of life in patients with breast cancer or head and neck cancer receiving radiotherapy.

**Supplementary Information:**

The online version contains supplementary material available at 10.1186/s13014-023-02363-9.

## Introduction

Radiation therapy (RT) is the standard treatment for most patients with head and neck cancer (HNC) or breast cancer (BC). The routine radiation dose of RT for these patients is often more than 50 Gy either as a primary or postoperative treatment. Many studies have confirmed RT-induced skin toxicity is closely associated with total dose and fractionation schedule [1, 2]. Radiation dermatitis (RD) occurs in approximately 95% of patients receiving radiotherapy [3]. Generally, the majority of acute RD can be recovered, but late RD is progressive and often irreversible [1, 4]. Prophylactic and early management for acute RD not only decrease the severity of skin effects, but also avoid the incidence of late RD which may significantly reduce patient quality of life (QoL) [1, 5]. Many pharmacological and non-pharmacological topical treatment for RD prophylaxis and management have shown great promise, but there is no consensus to date regarding the “gold standard” intervention [6].

Natural and miscellaneous agents are widely recommended by several guidelines for treatment of RD, such as Vitamin E, hyaluronic acids, aloe vera [7, 8]. In recent years, preclinical studies found miscellaneous agents containing linoleic acid and linolenic acid were effective in mitigating trans-epidermal water loss, skin erythema, melanin formation, and subcutaneous blood flow [9, 10]. Linoleic acid and linolenic acid can reduce mechanically release of chemical mediators through inhibition of NF-κB signaling which is closely related with radiation-induced tissue injury [11–14]. A liquid dressing containing linoleic acid and linolenic acid (Sanyrene, LABORATOIRES URGO, France) has been used to prevent the occurrence of pressure ulceration [15, 16]. However, to our knowledge, there is not yet any randomized controlled studies to evaluate the effects of Sanyrene for preventing RD in patients receiving radiotherapy. Therefore, the aim of this study was to conduct a randomized control trial to investigate the prophylactic effects of Sanyrene versus control intervention (DaBao, a cream containing hyaluronic acid and Vitamin E) for acute RD in patients with breast cancer or head and neck cancer receiving radiotherapy.

## Materials and methods

### Patient selection

The design of this study was a randomized controlled trial comparing Sanyrene with control intervention in 102 HNC and BC patients receiving radiation therapy. Patients were consecutively enrolled from June 2020 to July 2021 at department of radiation oncology in Xi’an, China. This study (Trial Registration: ChiCTR2100050910) was approved by the medical ethics committee of the first affiliated hospital of the air force medical university. Patients with age ≥ 18 years, ECOG of 0 or 1 and a pathologic diagnosis of HNC and BC receiving radiotherapy (≥ 50 Gy) were eligible. Exclusion criteria were prior RT to the intended field, palliative RT with dose < 50 Gy, receiving breast conserving surgery in BC patients, receiving anti-EGFR monoclonal antibody in HNC patients, pre-existing grade >1 skin toxicity, cellulitis, autoimmune skin disease or incompletely healed wound at intended site. Patients were also excluded if they had known allergic reaction towards any ingredient of Sanyrene, Vitamin E or hyaluronic acid were not able to consent. Receiving concurrent chemotherapy was not an exclusion criterion.

### Randomization

After completion of informed consent, participants were randomized to either the intervention arm (Sanyrene) or the control intervention arm in a ratio of 1:1. The randomization procedures were carried out by sealed enveloping from the central office of the Clinical Trials Centre. Blocked randomization was performed using permuted block with a block size of n = 4. Patients were stratified by the presence of cancer site. Only the statistician and the study coordinator knew the block structure, and the statistician and the study coordinator had no clinical involvement during the trial.

### Treatment

RT dose for patients with BC was 50 Gy/25 fractions delivered over 5 weeks. Most patients (88.2%) received 3-dimensional conformal radiation therapy (3D-CRT), and 6 patients (11.8%) received volumetric modulated arc radiation therapy (VMAT). To elevate skin dose, all patients were treated with 5 mm bolus over chest wall before 15 fractions of radiotherapy. For postoperative HNC patients, RT dose at 2 Gy/fraction was administrated once daily with five fraction per week up to total dose of 50-55 Gy. If radical radiotherapy was administrated, patients would receive up to a total dose of ≥ 66 Gy RT with cisplatin-based concurrent chemoradiotherapy (CCRT). Patients with high-risk factors of postoperation, such as positive margin and extracapsular spread in lymph node metastasis, received CCRT. The concurrent chemotherapy regimen was triweekly 80-100 mg/m^2^ cisplatin givens on days 1, 22, and 43.

For the Sanyrene arm and control arm, patients were asked to start topical application of Sanyrene and control intervention cream respectively on the area of skin being irradiated at the onset of radiotherapy, twice a day up to 14 days post treatment. Sanyrene is a liquid dressing with characteristics of oily, colorless and fragrance of fennel. Control intervention is a white cream with fragrance. If moist desquamation occurred, these interventions would be stopped, then treating nurses applied epidermal growth factor solution and Silver Sulphadiazine impregnated Hydrocolloid Dressing 10 cm×12 cm until the wound healed.

### Endpoint evaluation

All patients completed a range of questionnaires by interview every week from the start of radiotherapy treatment until four weeks after the completion of radiotherapy. The acute radiation morbidity scoring criteria of radiation therapy oncology group (RTOG) was used to grade provider-assessed toxicities. The primary endpoint was incidence rate of ≥ grade 2 skin toxicity, including tender or bright erythema, patchy moist desquamation and moderate edema [17]. Weekly assessments were conducted with blind by two radiation oncology nurses whose did not involve in the study from the initial treatment up to four weeks post radiation treatment. If consensus was not reached, a third nurse would assess the skin toxicity again and consensus meetings were conducted to resolve the disagreements.

The secondary endpoint of this study was dermatitis-related Qol which was usually evaluated by the Skindex-16 (SD-16) module. SD-16 comprises an analogue scale (0 = never bothered to 6 = always bothered) to categorize patient response, including emotion, symptoms and functioning [18, 19]. Patients completed the SD-16 assessment every week from the beginning of radiation treatment up to four weeks after end of radiation treatment. Numerical Rating Scale (NRS) with a consecutive scale (0–10) was used to screen the skin pain relief of patients every week from beginning of radiation treatment to four weeks after end of radiation treatment.

### Statistical analysis

According to our preliminary research results, the incidence rate of ≥ 2 grade RD in the control group is 65.6%. We estimated that approximately 102 patients would need to be randomized in a 1:1 ratio, with 51 patients in each group. This is to detect a difference in the incidence rate of ≥ grade 2: 65.6% in the control intervention group vs. 36.0% in the Sanyrene group. The estimation assumes a power of 80%, a two-sided significance level of 0.05, and an anticipated dropout rate of about 8%. An interim analysis was performed when reaching approximately half the sample size, the O’Brien–Fleming type boundary (alpha of 0.003) was used for early trial stoppage.

**Fig. 1:**
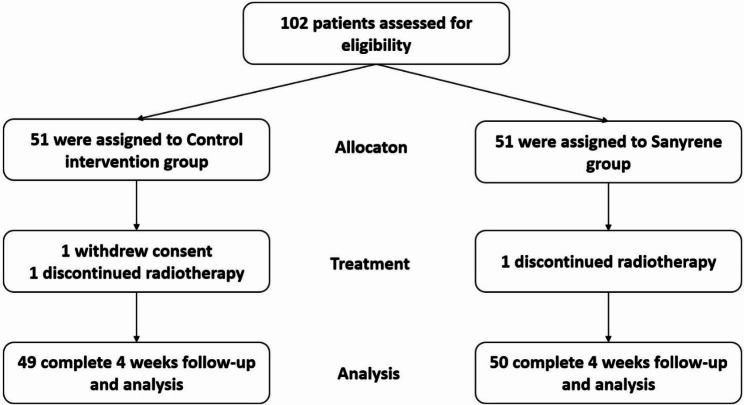
CONSORT Diagram

Baseline characteristics of patients were displayed in the intention-to-treat trial populations (see the Fig. 1 study flow chart). If the continuous variables conform to normal distribution after checking via the 1-Sample K-S test, the mean ± standard deviation is used to describe their concentration and dispersion trend, and the two independent sample t test is used for comparison between groups. If not, the median (quartile, lower quartile and upper quartile) would be used to describe their trends, Wilcoxon Rank Sum test for continuity variables and Pearson Chi-square test or continuity correction Chi-square test for categorical variables (shown by frequency and percentage) were used to compare the difference between the two groups. Efficacy analyses were performed in patients whose completed radiotherapy and 4 weeks follow-up. Kaplan–Meier curve was used to present time-to-≥grade 2 skin toxicity, and the two groups were compared by log-rank tests. Cox proportional-hazards model was used to calculate the hazard ratios and 95% confidence interval when treatment was considered as a single covariate, and the proportional-hazards assumption was tested with Schoenfeld residuals. The Statistical Package for Social Sciences (SPSS version 24.0, IBM, USA) software was used for statistical analyses. A two-tailed statistical probability of *p* < 0.05 was considered as significant.

## Results

### Patient characteristics

From May 2020 to September 2020, a total of 102 eligible patients were randomly assigned into the study. One patient in control intervention group withdrew consent and 1 patient failed to complete radiotherapy. One patient in Sanyrene group was excluded from final analysis due to incomplete radiotherapy. Ultimately, 99 patients were included in the final analysis (Fig. 1).

Patient characteristics were well balanced in both arms (Table 1). The median age of the whole cohort was 51 years old (range: 19–66 years). The median radiation dose of whole cohort were 50 Gy (range: 50-72.6 Gy). Of all patients included in final analysis, 65 (65.7%) were female and 34 (34.3%) were male. A total of 46 patients (46.5%) received 3D-CRT, while the others received VMAT. Only 13 patients (13.1%) with head and neck cancer received concurrent chemotherapy.

**Table 1:** Baseline characteristics of patients [Median (range)/n (%)]

Variables	Control intervention (n = 49)	Sanyrene (n = 50)	*p*
Median BMI(kg/m^2^)	23.0(17.8, 34.4)	23.9(16.2, 29.4)	0.499
Median Total dose (Gy)	50.0 (50.0, 72.6)	50.0 (16.0, 72.6)	0.327
BMI(kg/m^2^)			0.164
<18.5	1(2.0)	2(4.0)	
18.5–23.9	29(59.2)	24(48.0)	
24.0-27.9	13(26.5)	22(44.0)	
≥28.0	6(12.2)	2(4.0)	
Age (years)			0.270
≤50	26(53.1)	21(42.0)	
>50	23(46.9)	29(58.0)	
Sex			0.726
Male	16(32.7)	18(36.0)	
Female	33(67.3)	32(64.0)	
Smoke			0.248
No	49(100.0)	47(94.0)	
Yes	0(0)	3(6.0)	
Drink			0.986
No	47(95.9)	49(98.0)	
Yes	2(4.1)	1(2.0)	
ECOG			0.484
0	24(49.0)	28(56.0)	
1	25(51.0)	22(44.0)	
Tumor			0.761
Breast cancer	26(53.1)	25(50.0)	
Head and neck cancer	23(46.9)	25(50.0)	
Radiation Technique			0.757
3D-CRT	22(44.9)	24(48.0)	
VMAT	27(55.1)	26(52.0)	
CCRT			0.736
No	42(85.7)	44(88.0)	
Yes	7(14.3)	6(12.0)	

BMI, body mass index; 3D-CRT, 3-dimensional conformal radiation therapy; VMAT, volumetric modulated arc radiation therapy; CCRT, concurrent chemotherapy

### Efficacy

**Fig. 2:**
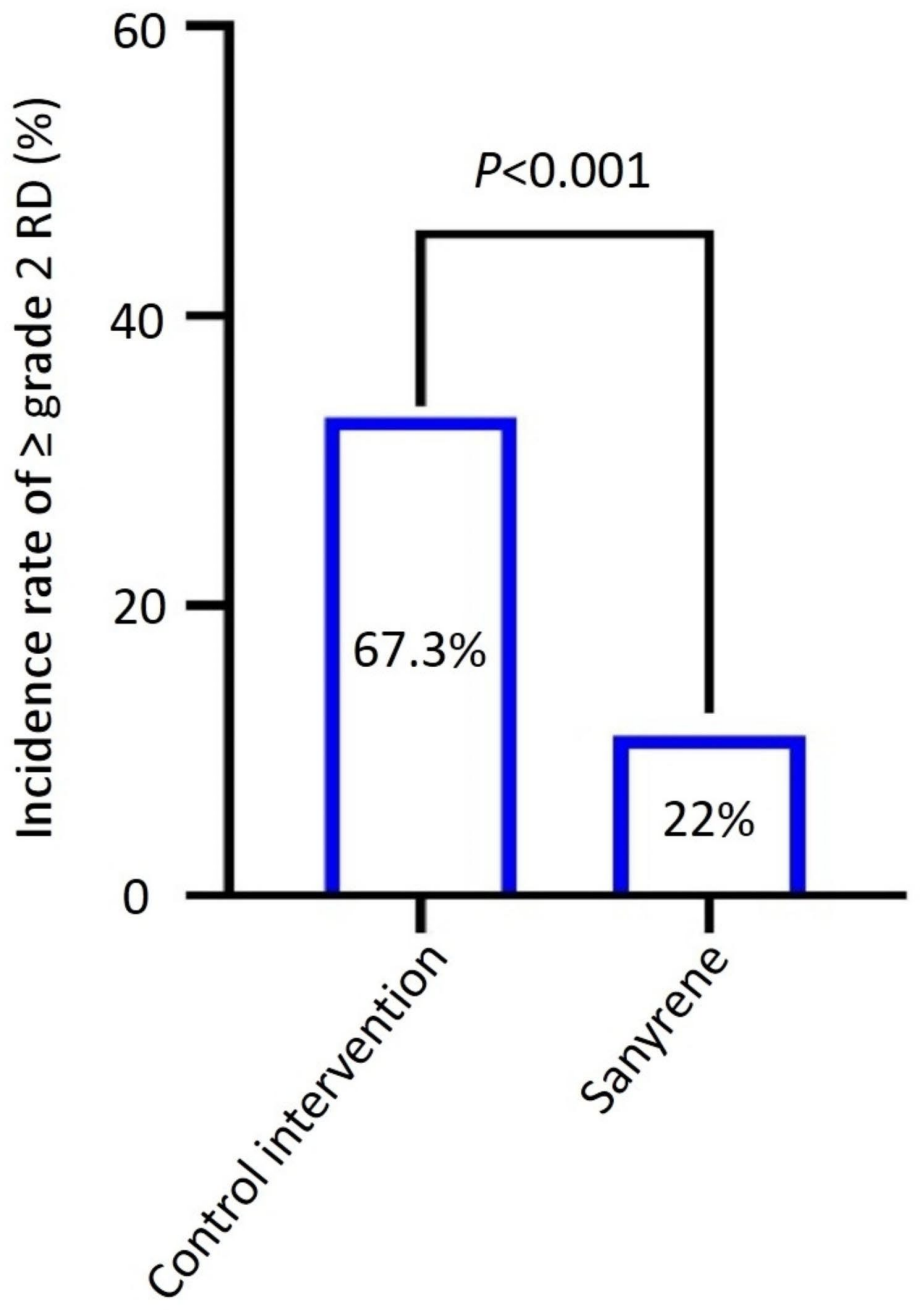
Incidence rate of ≥ grade 2 of radiation dermatitis in two treatment group

**Fig. 3:**
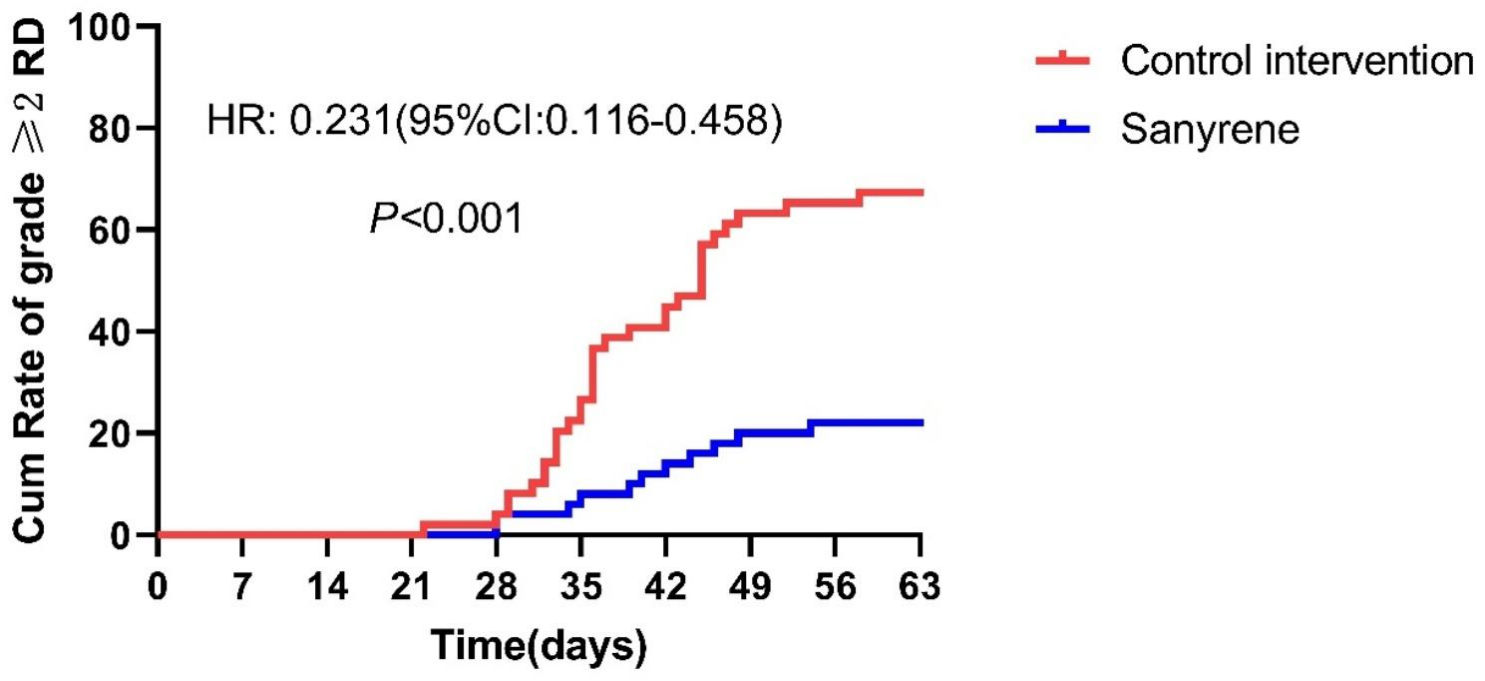
Incidence rate of time to ≥ grade 2 of radiation dermatitis between two groups

All patients completed their follow-up records at 4 weeks post-radiotherapy. Of all patients, 55 patients (55.5%) experienced grade 1 skin toxicity, 30 patients (30.3%) experienced grade 2 toxicity, and 14 patients (14.1%) experienced grade 3 skin toxicity during the whole treatment and follow-up period. None of patients in this study experienced grade 4 RD. The incidence rate of ≥ grade 2 RD was 22% (95% confidence interval (CI), 12–36.3%) in Sanyrene group, as compared with 67.3% (95% CI, 52.3–79.6%) in the control intervention group (*P*<0.001) (Fig. 2). The incidence of grade 3 RD was 20.4% and 8.0% in control intervention group and Sanyrene group, respectively (*p* = 0.076) (Table 2). The median time to reach grade 1 RD was 29 days in Sanyrene group and 28 days in control intervention group (HR:0.473, 95%CI:0.313–0.714, *P*<0.001) (Figure S1). Patients in Sanyrene group had a longer median time to reach ≥ grade 2 RD compared to those in control intervention group, with hazard ratio of 0.231(95%CI:0.116–0.458, *p* < 0.001) (Fig. 3). Although there was a trend to prolong the time to grade 3 RD in Sanyrene group, statistical difference was not achieved between two groups (Figure S2). Cox regression analysis showed total dose of radiation (continuous variable)(HR:1.034,95%CI:1.005–1.063,*p* = 0.019) and treatment group (Sanyrene vs. control intervention) (HR:0.247, 95%CI:0.124–0.493, *P*<0.001) were independently prognostic factors for incidence of ≥ grade 2 RD (Table 3). Subgroup analyses indicated patients in Sanyrene group had lower incidence rate of ≥ grade 2 RD than those in control intervention group (HNC: 32.0% vs.82.6%, *P*<0.001; BC: 12.0% vs. 53.9%, *p* = 0.002) (Table S1).

**Table 2:** RTOG skin toxicities in both groups [n (%)]

Group	Grade 1	Grade 2	Grade 3
Control intervention (n = 49)	16(32.7)	23(46.9)	10(20.4)
Sanyrene (n = 50)	39(78.0)	7(14.0)	4(8.0)
*p*	< 0.001	< 0.001	0.076

RTOG, Radiation Therapy Oncology Group

**Table 3:** Univariate and multivariate analysis for grade 2 skin toxicity

Variables	Univariate			Multivariate		
	HR	95%CI	*P*	HR	95%CI	*P*
BMI(kg/m^2^)	1.074	0.978–1.179	0.134			
Total dose (Gy)	1.041	1.012–1.071	0.005	1.034	1.005–1.063	0.019
Treatment Group (Sanyrene vs. Control intervention)	0.231	0.116–0.458	< 0.001	0.247	0.124–0.493	< 0.001
Age (> 50 vs. ≤50)	1.117	0.617–2.023	0.714			
Sex (female vs. male)	0.588	0.325–1.066	0.080			
Smoke (yes vs. no)	1.448	0.350–5.987	0.609			
Drink (yes vs. no)	3.038	0.937–9.855	0.064			
ECOG (1 vs. 0)	0.660	0.359–1.211	0.180			
Tumor (HNC vs. BC)	1.731	0.942–3.178	0.077			
RT (VMAT vs. 3D-CRT)	2.001	1.060–3.779	0.032			
CCRT(yes vs. no)	1.411	0.655–3.040	0.379			

HNC, head and neck cancer; BC, breast cancer; 3D-CRT, 3-dimensional conformal radiation therapy; VMAT, volumetric modulated arc radiation therapy; CCRT, Concurrent chemotherapy

**Fig. 4:**
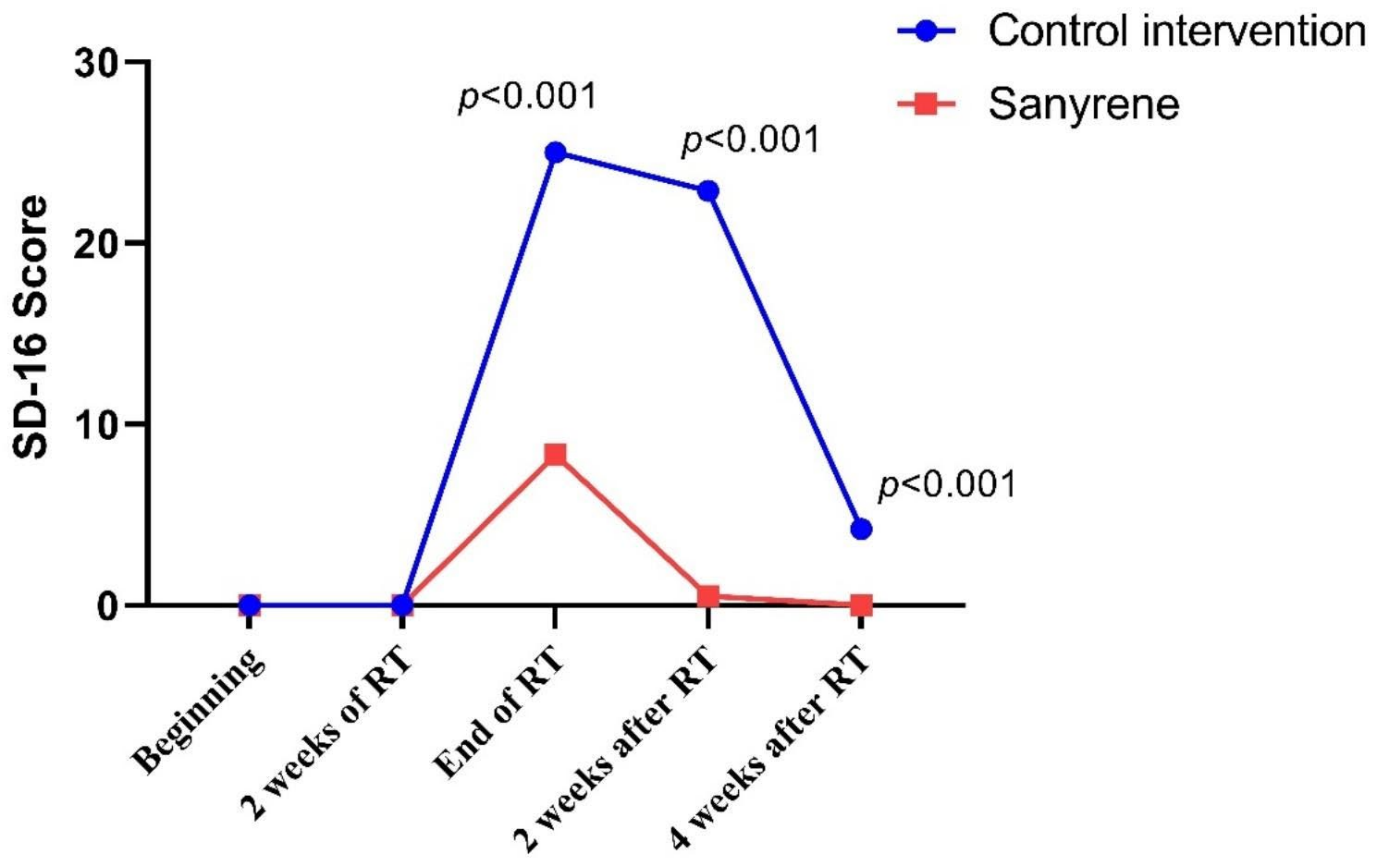
Mean score of SD-16 between two groups in whole treatment process

Mean score of SD-16 were much higher in control intervention group than Sanyrene group at end of RT (25 vs.8.3), 2 weeks after RT (22.9 vs. 0.5) and 4 weeks after RT (4.2 vs.0), with significantly statistical difference between two groups (Fig. 4). There were not significant differences in SD-16 score between patients receiving concurrent chemotherapy and patients not receiving concurrent chemotherapy (Figure S3). During entire treatment course and follow-up, 16 of 99 patients (16.2%) had no pain, 77 of 99 patients (73.7%) had mild pain (NRS score:1–3) and 10 of 99 patients (10.1%) had moderate pain (NRS score:4–6). In the Sanyene group, more patients achieved an NRS score of 0 compared to the control intervention group. (26% vs. 6.1%, *p* = 0.007) (Figure S4).

## Discussion

To our knowledge, this is the first study to identify the prophylactic effect of Sanyrene- a liquid dressing with linoleic acid and linolenic acid mixture in radiotherapy induced dermatitis. In this study, we found Sanyrene significantly reduced the incidence rate of ≥ grade 2 RD when compared to control intervention. Moreover, patients in Sanyrene group had better Qol and more mild pain than those in control intervention group during the whole RT process up to 4 weeks after end of RT. As there is no consensus on the standard intervention, our study is an evidence to supplement the strategy of RD prophylaxis and management.

Barrier films and dressings are kind of treatment methods for acute RD, but there is lack of consensus recommendation among guidelines for use of these methods. A single-blinded, randomized controlled study reported a silicone-based film dressing could reduce 41% and 49.4% risks of developing grade 2 and 3 skin toxicity compared to control arm in patients with head and neck cancer receiving radiotherapy, but skin related Qol was not improved by this dressing [20]. Silver based dressings have also been investigated to prevent acute RD, but results remain controversial. A phase 2 study reported silver based dressing improved pain control instead of preventing acute RD as compared to control group in patients with head and neck caner receiving RT [21]. However, other studies found silver based dressing was effective in reducing RD in patients undergoing RT to permeum and lower gastrointestinal sites [22, 23]. Sanyrene as liquid dressing is extensively used to prevent pressure ulceration and xeroderma [15]. This liquid dressing containing linoleic acid and linolenic acid was easy to apply and particular emphasis was given on no adverse events due to this product in this study. Our study found Sanyrene had simultaneous effects on preventing serious RD and improving skin related Qol in patients with BC and HNC receiving radiotherapy.

Many studies reported linoleic acid and linolenic acid were benefit for skin by attenuating cutaneous inflammation through the competition with the inflammatory arachidonic acid and the inhibition of proinflammatory eicosanoid production [10, 24, 25]. Therefore, Sanyrene may reduce RT induced inflammation process through above mechanism. Besides, preclinical study suggested blocking arachidonic acid by drug might reduce skin damage after RT [26]. Regrettably, several studies reported celecoxib, an inhibitor of COX-2 which inhibits the conversion of arachidonic acid to prostaglandin E, did not has effectively relieve radiation and ultraviolet B induced dermatitis [27, 28]. Corticosteroids with natural anti-inflammatory properties have been extensively investigated the efficacy of RD prophylaxis. Clinical trials reported topical corticosteroids reduced the frequency of acute RD in patients receiving radiotherapy [29–31]. A study reported mometasone furoate combined with emollient cream significantly reduced acute RD compared to emollient cream alone in patients with breast cancer receiving RT [29]. Ho et al. conducted a randomized controlled study to investigate the efficacy of mometasone furoate in reducing high grade acute RD in breast cancer patients receiving RT, and revealed mometasone furoate significantly reduced the incidence of maximum skin toxicities and prolonged time to development of grade 3 dermatitis compared to control cream [30]. Another double-blinded, phase 3 trial reported mometasone furoate failed to provide better remission rate of acute RD than control arm, but improved the dermatitis related Qol [31]. Based on these evidences, many guidelines recommended corticosteroids to treat acute RD [6]. However, it is worth noting that corticosteroids can cause thinning of the skin which can potentially cause skin dehydration [32]. In the future, we will design a randomized controlled study to compare Sanyrene with corticosteroids in preventing RD.

There were some limitations in this study. Firstly, it was difficult to blind physicians or patients in this trial because Sanyrene and control intervention (a cream containing Vitamin E and hyaluronic acid) had different properties. To reduce the confounding bias as much as possible, we chose two nurses who were not involved in the study to assess the grade of skin toxicity according to acute RTOG criteria. If consensus was not reached, the third nurse would assess the skin toxicity again and consensus meetings were conducted to resolve the disagreements. These nurses accepted training of toxicity assessing before beginning of the study. Therefore, we did blind the assessors of RD grade for trying to avoid bias. Secondly, subgroup analysis found Sanyrene effectively prevented incidence rate of ≥ grade 2 RD in patients with BC and HNC respectively, but bias was unavoidable due to small sample in each subgroup. Especially for head and neck cancer, concurrent chemoradiotherapy which could improve incidence of RD was not a rigorous inclusion criteria. Therefore, randomized controlled studies aiming to single disease should be conducted to confirm the efficacy of Sanyrene in preventing RD.

## Conclusion

This trial suggests that Sanyrene is effective on preventing serious RD and improving skin related Qol in patients with breast cancer and head and neck cancer receiving radiotherapy. However, these results should be further validated by trials with rigorous design.

### Electronic supplementary material

Below is the link to the electronic supplementary material.

Supplementary Material 1

Supplementary Material 2

Supplementary Material 3

Supplementary Material 4

Supplementary Material 5

## Data Availability

All data generated or analyzed during this study are included in this article. Further enquiries can be directed to the corresponding author.
