# Supplementary material for: A blinded-endpoint, randomized controlled trial of Sanyrene with natural active ingredient for prophylaxis of radiation dermatitis in patients receiving radiotherapy
Source: Radiat Oncol. 2023 Oct 27;18:174. doi: 10.1186/s13014-023-02363-9 (PMC10604398; doi:10.1186/s13014-023-02363-9)
Supplement: Supplementary file 1 — Supplementary Material 1 [file 13014_2023_2363_MOESM1_ESM.docx]

| Variables | Control intervention (n=49) | Sanyrene (n=50) | *p* |
| --- | --- | --- | --- |
| HNC (RTOG ≥2) | 19/23(82.6%) | 8/25(32.0%) | <0.001 |
| BC (RTOG ≥2) | 14/26(53.9%) | 3/25(12.0%) | 0.002 |

Table S1 incidence rate of grade 2 skin toxicity in subgroup analysis

HNC, head and neck cancer; BC, breast cancer
