# Supplementary figures and images for: A blinded-endpoint, randomized controlled trial of Sanyrene with natural active ingredient for prophylaxis of radiation dermatitis in patients receiving radiotherapy
Source: Radiat Oncol. 2023 Oct 27;18:174. doi: 10.1186/s13014-023-02363-9 (PMC10604398; doi:10.1186/s13014-023-02363-9)

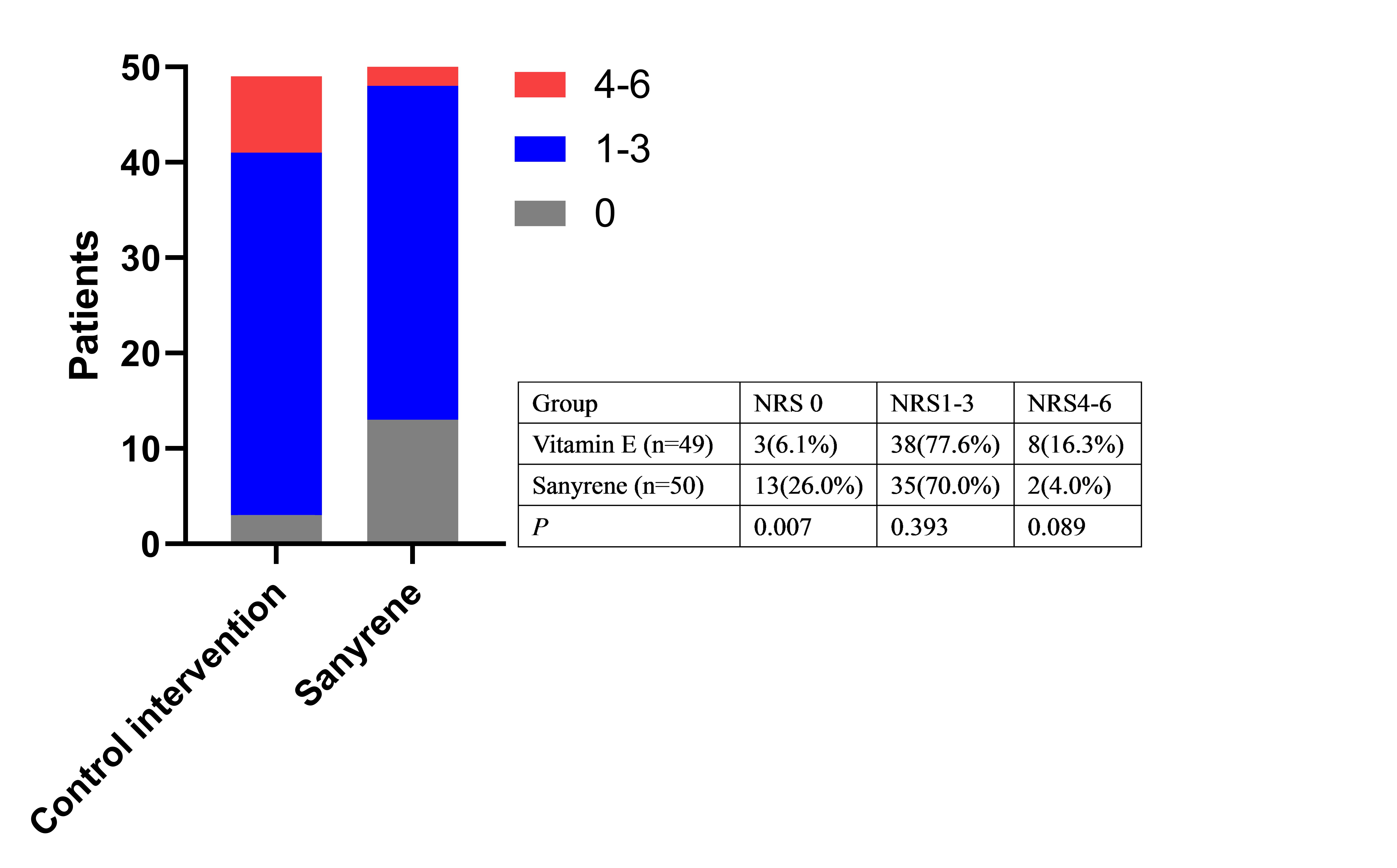

Supplement: Supplementary file 2 — Supplementary Material 2 [file 13014_2023_2363_MOESM2_ESM.jpg]

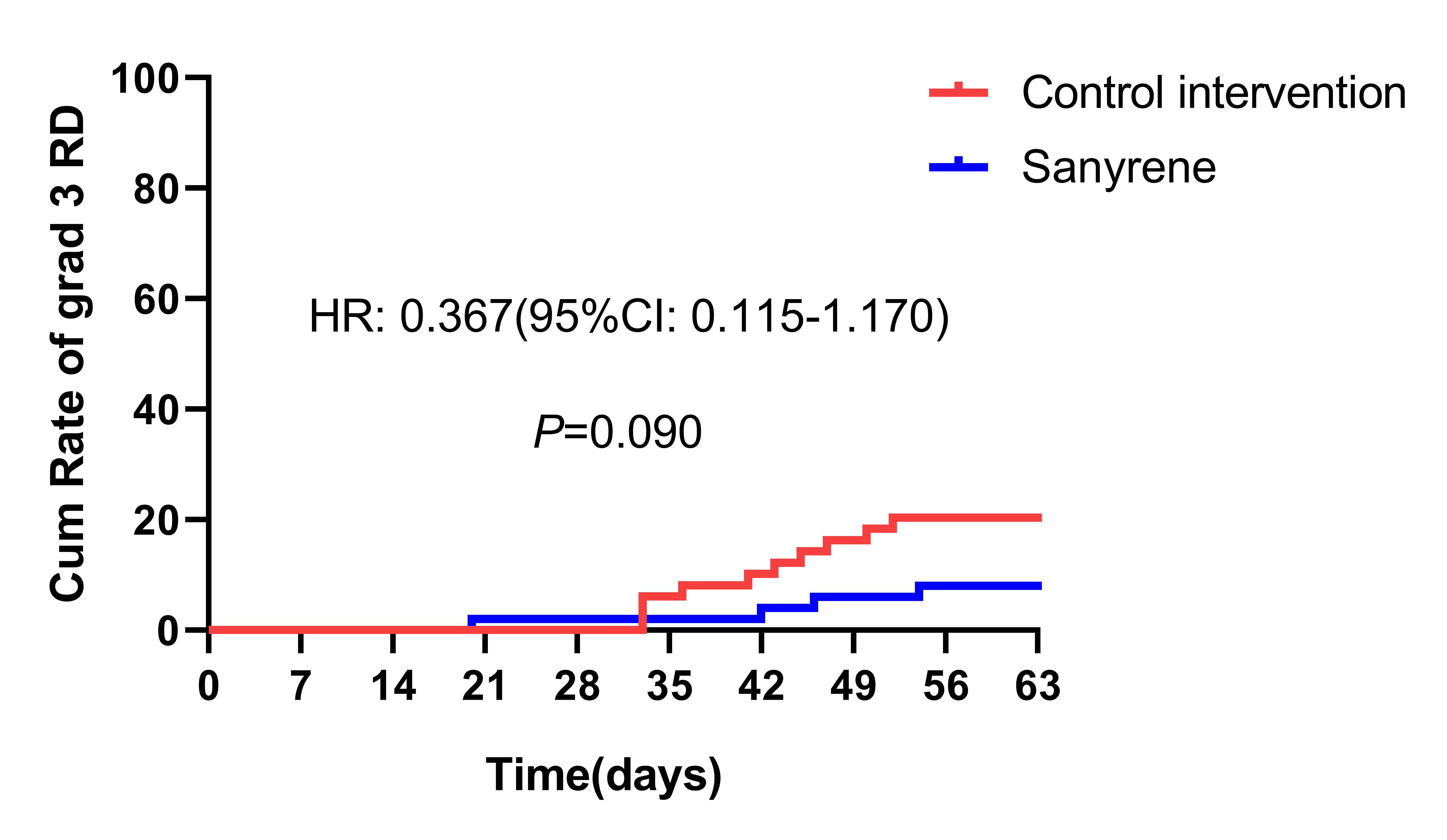

Supplement: Supplementary file 3 — Supplementary Material 3 [file 13014_2023_2363_MOESM3_ESM.jpg]

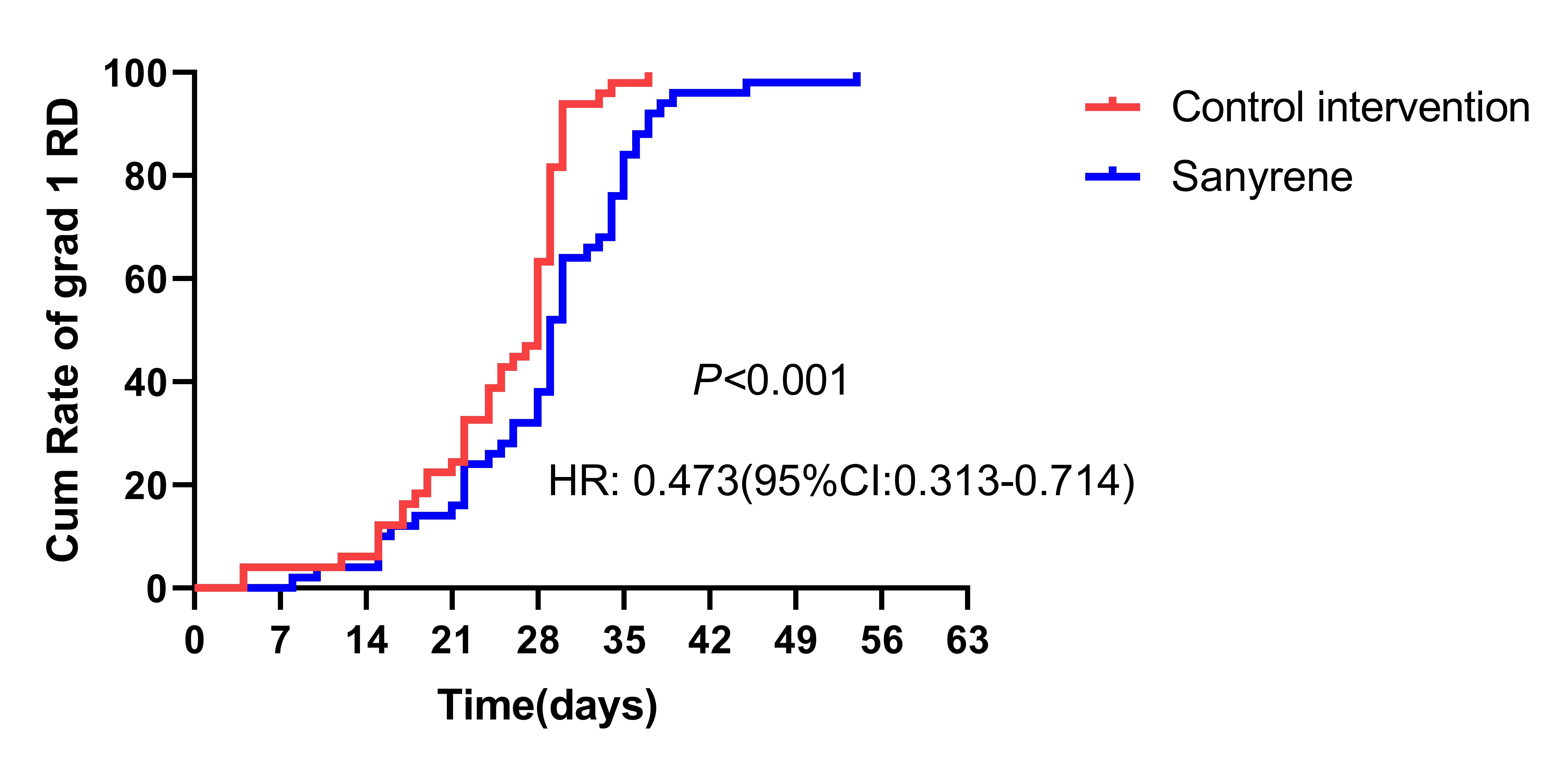

Supplement: Supplementary file 4 — Supplementary Material 4 [file 13014_2023_2363_MOESM4_ESM.jpg]

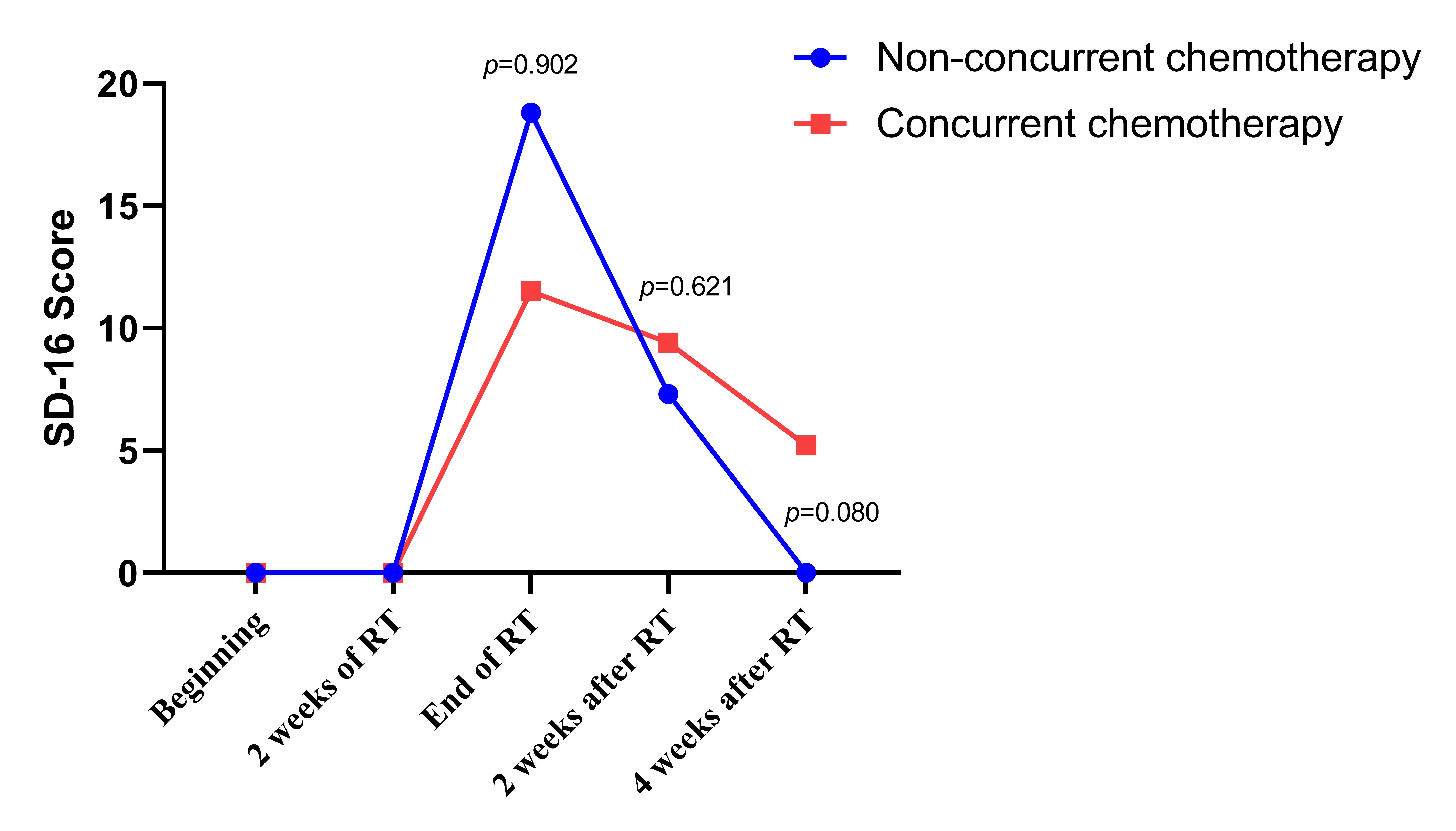

Supplement: Supplementary file 5 — Supplementary Material 5 [file 13014_2023_2363_MOESM5_ESM.jpg]
